# Is it okay to use a commercially available AST for *Burkholderia cepacia* complex?

**DOI:** 10.1128/jcm.01871-25

**Published:** 2026-05-14

**Authors:** April M. Bobenchik, Priyanka Uprety

**Affiliations:** 1 Penn State Hershey Medical Center and College of Medicine, Hershey, Pennsylvania, USA; 2 Quest Diagnostics, Clifton, New Jersey, USA; Vanderbilt University Medical Center, Nashville, Tennessee, USA

## Abstract

In the article published by K. L. Shier, G. Gonzales, D. A. G. Perez, J. R. Bao, et al. (J Clin Microbiol 64:e00484-25, 2026, https://www.doi.org/10.1128/jcm.00484-25), the authors evaluated the performance of the Vitek 2 (bioMérieux), Sensititre (Thermo Fisher), and Etest (bioMérieux) Antimicrobial Susceptibility Testing (AST) methods compared to reference broth microdilution for 46 reference *Burkholderia cepacia* complex (BCC) isolates. This study reports on several of the most widely used commercial AST systems across laboratory settings, including commercial reference laboratories, and concludes that Sensititre may be a viable commercial option when susceptibility testing is warranted. Here, we review the reproducibility and performance characteristics of various AST methods for BCC, highlight the challenges of performing AST, and discuss the future of susceptibility testing for this organism group.

## COMMENTARY

*Burkholderia cepacia* complex (BCC) is an opportunistic pathogen that can cause severe infections in cystic fibrosis (CF) and immunocompromised patients (1). There are multiple species belonging to the BCC; however, *B. cenocepacia*, *B. cepacia*, and *B. multivorans* are the three most common species isolated from clinical specimens (2). Performing antimicrobial susceptibility testing (AST) for nonfermenting gram-negative bacilli can be challenging, and this is particularly true for BCC. In recent years, both the Clinical and Laboratory Standards Institute (CLSI) and the European Committee on Antimicrobial Susceptibility Testing (EUCAST) have released guidance that indicates performing routine AST to guide the therapeutic management of patients with BCC infections is not recommended (3, 4). Furthermore, CLSI states that the only reproducible method for testing BCC is frozen reference broth microdilution (rBMD) and has removed disk diffusion (DD) and agar dilution (AD) methods for this organism group (3).

However, most clinicians desire accurate, reproducible AST results to guide therapeutic decisions, particularly for BCC due to their multiple intrinsic and acquired resistance mechanisms, making them difficult to treat empirically. Many laboratories use commercial methods, such as automated platforms, lyophilized broth microdilution panels, or gradient diffusion strips to perform AST, and as such, it is important to understand if these methods perform better than those evaluated by CLSI and EUCAST. In the article entitled “Performance of the Vitek 2, Sensititre, and Etest Antimicrobial Susceptibility Testing systems for isolates of *Burkholderia cepacia* complex,” the authors reported categorical (CA) and essential agreement (EA) that ranged from 44% to 95% across platforms and tested antimicrobials (5). These findings are consistent with other published studies that have evaluated commercially available AST methods for the BCC (6–9). Shier et al.’s work is important because it evaluated three commercial ASTs, two of which, Vitek 2 and Sensititre lyophilized broth microdilution panels, have not been systematically studied with BCC isolates, and found that Sensititre performed the best of the three methods evaluated, despite the majority of the antimicrobials not meeting acceptance criteria, and suggests that Sensititre may be the best commercially available option for clinical laboratories, if testing were to be performed.

However, a limitation of Shier et al.’s study was the lack of robust reproducibility testing. No reproducibility studies were performed for Sensititre, and Vitek 2 was reproducible, but the study was very small, testing only three isolates. So, although Sensititre had a slightly better agreement with rBMD compared to Vitek 2 and Etest, we do not know if the method is reproducible for testing BCC isolates. Therefore, it is unknown if it could be used as an alternative to rBMD. A review of method reproducibility and performance for BCC isolated from both CF and non-CF patients is highlighted below.

rBMD is considered the “gold standard” for aerobic bacterial AST and is the CLSI reference method for aerobic bacteria (3, 10). It is also the comparator method used to evaluate the performance of other AST methods. CLSI specifically recommends “frozen” rBMD for AST of BCC to make the distinction that the reference method is not the same as commercially prepared lyophilized BMD panels like Sensititre or MicroScan. CLSI recommends rBMD because it was found to be the only reproducible AST method for BCC. Depending on the study, reproducibility testing for rBMD ranged from 70.3% to >95% (6–8), Table 1. In a study published by Wootton et al., evaluating 155 BCC isolates from CF patients, reproducibility ranged from 70.3% to 84.5% (8). A study by Huse et al., evaluating 100 BCC isolates from CF patients, showed higher reproducibility, ranging from 82% to 97%, with two antimicrobials, minocycline (MIN) and meropenem (MEM), achieving 97% (7). A follow-up study by Jorth et al., which included both CF and non-CF BCC isolates, demonstrated comparable results with reproducibility ranging from 93% to 99%, with two additional antimicrobials, levofloxacin (LVX) and trimethoprim-sulfamethoxazole (TMP-SMX), meeting acceptance criteria (6). Reproducibility was the highest for the non-CF BCC isolates, ranging from 96% to 100% for all antimicrobials tested.

**TABLE 1:** Comparison of methods and antimicrobials that met acceptable criteria (≥95%) for reproducibility

Study	rBMD	AD	DD (within 3 mm)	Etest	MicroScan	Sensititre	Vitek 2
Shier et al. (5) 3 randomly selected isolates	–^*a*^	–	–	–	–	–	CAZ MEM MIN LVX TMP-SMX
Jorth et al. (6) 100 CF 105 Non-CF	MEM MIN LVX SXT	–	–	–	–	–	–
Huse et al. (7) 100 CF	MEM MIN	–	None	–	CAZ MEM MIN LVX CIP	–	–
Wootton et al. (8) 155 CF	None	–	TMP-SMX	–	–	–	–

^*a*^ "–”, no data for that AST method in that study.

In contrast to rBMD, DD was not reproducible with a range of 64–86% for all antimicrobials tested (7). Wootton et al., who tested only three antimicrobials, TMP-SMX, MEM, and ceftazidime (TAZ), found DD to be reproducible for only TMP-SMX (97.4%) (8). More importantly, when DD was compared to rBMD, performance was poor overall (6–9), Table 2. Huse et al. demonstrated acceptable CA for only MEM (7), whereas Fehlberg et al. showed acceptable performance with a CA of >91% for CAZ, MEM, and MIN (9). Jorth et al. investigated whether the manufacture of agar media had an effect on DD performance and whether non-CF isolates performed better and found that DD did not meet the acceptance criteria for any of the antimicrobials tested, regardless of the media manufacturer used. When evaluating CF vs non-CF isolates, the non-CF isolates overall had fewer categorical errors than the CF isolates but still did not meet acceptance criteria. The type and rate of errors varied by antimicrobial and by isolate source, and the authors concluded that neither media type nor isolate source had an impact on meeting acceptance criteria (6). Due to its poor reproducibility and agreement with rBMD, DD is not a recommended method for BCC AST.

**TABLE 2:** Comparison of methods and antimicrobials that met acceptable criteria for EA and CA with rBMD^*a*^

Study	MIC/category	AD	DD	Etest	MicroScan	Sensititre	Vitek 2
Shier et al. (5) 24 CF 22 Non-CF	EA	–	–	LVX MEM	–	CAZ LVX	TMP-SMX
	CA	–	–	None	–	None	TMP-SMX
Jorth et al. (6) 100 CF 105 Non-CF	EA	LVX	N/A	None	–	–	–
	CA	LVX	None	LVX	–	–	–
Huse et al. (7) 100 CF	EA	–	N/A	–	None	–	–
	CA	–	MEM	–	None	–	–
Wootton et al. (8) 155 CF	EA	None	N/A	None	–	–	–
	CA	N/A	None	None	–	–	–
Fehlber et al. (9) 47 CF 35 Non-CF	EA	CAZ LVX MEM MIN TMP-SMX	N/A	LVX MEN MIN	–	–	–
	CA	CAZ MEM TMP-SMX	CAZ MEM MIN	CAZ MEM MIN TMP-SMX	–	–	–

^*a*^ "–”, no data for that AST method in that study.

AD is a CLSI reference method for anaerobic bacteria and some fastidious organisms (11). Each agar plate contains a different concentration of an antimicrobial, and the MIC corresponds to the first plate in the dilution series where growth is inhibited. Three studies evaluated AD compared to rBMD for BCC isolates, Table 2. In a study by Jorth et al., AD was found to have acceptable EA and CA with rBMD for only one antimicrobial, LVX, (96%), and no antimicrobials met the acceptance criteria when CF isolates were evaluated individually (6). Many of the errors were due to lower MICs observed with AD, resulting in false susceptibility. Wootton et al. evaluated AD with rBMD at two incubation temperatures, 35°C and 30°C, and found EA to range from 32.9% to 80.7% at 35°C with no improvement in EA with incubation at 30°C. Wootton et al. also found that AD MICs were lower compared to rBMD MICs, which were attributed to the lower inoculum used (8). This is in contrast to the Fehlberg study, which showed EA >93% for all antimicrobials (CAZ, MEM, MIN, LEV, chloramphenicol [CAM], and TMP-SXT) tested except CAM (89%) and CA for three of six antimicrobials (MEM, 91.4%; CAZ, 97.5%; and TMP-SXT, 100%) (9). Unfortunately, reproducibility studies have not been published for AD; therefore, it is unknown if AD is as reproducible as rBMD.

It is important to note that AD differs from gradient diffusion. Gradient diffusion methods, including Etest (bioMérieux) and MTS (Liofilchem), are more similar to DD in that the antimicrobial diffuses out of the strip into the agar. Unlike DD, which contains a fixed concentration of an antimicrobial, gradient diffusion strips contain multiple dilutions along a gradient and can provide an MIC result. The same authors who evaluated DD and AD also evaluated the commercial gradient diffusion method, Etest. As seen with DD and AD, the Jorth, Wootton, and Shier studies showed poor CA for Etest, with only LVX meeting acceptable CA in the Jorth study (5, 6, 8). It was noted in this study that a proportion of isolates were too faint to read at 24 h and were read at 48 h following the Etest instructions for use for testing nonfermenting gram-negative bacilli (6). However, this was not noted in the Shier et al. study or the other previously published studies on Etest performance of BCC (5, 8, 9). Fehlberg et al. compared Etest to both rBMD and AD and showed >90 EA for all antimicrobials except CAZ for AD and CAZ, CAM, and TMP-SMX for rBMD (9).

In addition to the recently published study by Shier et al., one other study has evaluated the performance of commercial AST devices using BCC isolates from CF patients. Huse et al. evaluated the MicroScan Walkaway system and showed strong reproducibility ranging from 88% to 100% (7). However, MicroScan concordance with rBMD was poor, with none of the antimicrobials meeting the acceptance criteria for agreement with rBMD. Of the commercial AST devices, Sensititre performed the best with EA ranging from 84.4% to 97.8%, and CA ranging from 68.9% to 88.9%. Vitek 2 EA ranged from 44.2% to 95.2%, and CA ranged from 58.1% to 92.9% (5). All three methods exhibited lower MICs compared to rBMD with an overall trend toward undercalling resistance.

In summary, all studies showed variable reproducibility (Table 1), including rBMD, and consistently showed poor agreement with DD and commercial methods (Table 2) (5–9). Of the MIC methods, the performance of AD was the most concerning because AD and rBMD are both CLSI reference methods and did not correlate with each other when testing a diverse, more resistant population of BCC isolates (6, 8). The poor correlation between AD and rBMD led to CLSI removing AD as a recommended method for BCC susceptibility testing in 2025, leaving only rBMD as a CLSI-recognized reference method for BCC. DD was removed in 2024 (3, 12). Despite rBMD being considered the “gold standard,” it is unknown if it is the correct method for determining the true MIC for BCC. However, we can say with a degree of certainty that rBMD has consistently demonstrated reproducible MICs, although not for all antimicrobials, and not consistently above 95%. The non-reference MIC method, Etest, has been evaluated across multiple studies and showed the most consistently acceptable performance for only two antimicrobials, LEV and MEM (5, 6, 9). Unfortunately, there are no reproducibility studies published for the Etest or other gradient diffusion methods with BCC isolates. To date, only one study has evaluated the performance of an automated method, focusing solely on BCC isolates, using MicroScan (7). This highlights the importance of the Shier et al. study because it fills a gap in the understanding of automated AST performance for BCC isolates. It was interesting to see that both MicroScan and Vitek 2 exhibited good reproducibility but showed poor correlation with rBMD (5, 7). In agreement with Shier et al., Sensititre lyophilized BMD performed the best of the commercial methods across published studies to date, but the lack of reproducibility data and the small data set make it difficult to confidently recommend this method as an alternative to rBMD. Taken together, all these studies reinforce the sentiment that there is no optimal method for BCC AST.

It is important to point out that all evaluated methods used at least in part standardized methods, including inoculum, media, and atmospheric conditions for incubation (10, 13, 14). Incubation temperatures were varied in one study, with no difference in outcome (8). Incubation length ranged from 18 to 20 h, with some studies extending to 24 or 48 h for isolates that showed poor growth (6, 9). Overall, testing BCC using standardized AST has proven very difficult. This is not unexpected due to the multiple mechanisms of resistance that BCC harbor. BCC are environmental organisms that have adapted to their harsh environment through a combination of intrinsic and acquired resistance. They have a modified lipopolysaccharide outer membrane, express fewer porins, and overexpress efflux pumps, making it very difficult for antibiotics to get into the cell. Additionally, they have target mutations conferring resistance to fluoroquinolones and express enzymes such as β-lactamases that hydrolyze β-lactams (15). Variable expression of these resistance mechanisms makes it extremely hard to achieve reproducible results using a standardized method. Moreover, isolates obtained from CF patients could have altered growth kinetics due to their adaptation to the lung microenvironment (16).

To conclude, rBMD has consistently shown to be the most reproducible method for testing BCC. However, despite the ability to achieve consistent MIC results, *in vitro* susceptibility testing cannot be correlated to clinical outcomes (17). Both CLSI and EUCAST have removed clinical breakpoints, not only because of poor correlation between methods, but also because of the lack of PK/PD data and clinical studies (3, 4). In the absence of clinical breakpoints, the use of AST for BCC to direct antimicrobial therapy selection should be strongly discouraged. However, the use of AST for BCC in basic research and drug discovery should continue. rBMD remains in the CLSI M100 as a recommended reference method for performing MIC testing to aid in the development of novel agents and to directly assess the *in vitro* activity of currently available newer agents, such as ceftazidime-avibactam (18) and cefiderocol (19). Finally, there is a need to explore new methods, whether it be non-standard phenotypic methods or genomic methods, to more accurately predict *in vivo* efficacy.
